# Quality of life of obese patients after treatment with the insertion of intra-gastric balloon versus Atkins diet in Sulaimani Governorate, Kurdistan Region, Iraq

**DOI:** 10.1016/j.amsu.2018.11.014

**Published:** 2018-11-24

**Authors:** Hiwa Omer Ahmed, Rajan Fuad Ezzat

**Affiliations:** a Professor in Metabolic and Bariatric Surgery, Senior Lecturer in College of Medicine, University of Sulaimani, Manager of Hospital for Endoscopic and Bariatric Surgery, Sulaimani City, Kurdistan, Iraq; b General Surgeon, Suliamani Teaching Hospital, Sulaimani City, Kurdistan, Iraq

**Keywords:** Obesity, Quality of life, Loss of weight, BioEnterics intra-gastric balloon, Atkins diet

## Abstract

**Background:**

Health-related quality of life of obese patients, before and after weight loss by insertion of BioEnterics Intra-gastric Balloon is studied widely. But the quality of life not related to comorbidity of the obese patients like mood, satisfaction with relationships, achieved goals, self-concepts, and self-perceived ability to cope with one's daily life is not studied on a wide scale.

**Aim:**

To evaluates the effect of the obesity on different aspects of life, and to evaluate the influence of weight loss after BIB insertion or Atkins diet on the quality of life regarding mood, satisfaction with relationships, achieved goals, self-concepts, and self-perceived ability to cope with one's daily life.

**Patients, materials, and methods:**

A prospective randomized study, from a total of 180 patients, 80 patients were selected to enroll in the work, over a period of 4 years from 2008 to 2012 in Hatwan private hospital and the private clinic, the closing date was 1st January 2013.

Approval for the current work was obtained from the Ethics Committee of University, College of Medicine. The work has been reported in line with the STROCSS criteria. The study was designed as a descriptive longitudinal study conducted on 40 patients who underwent intra-gastric balloon insertion compared to a matched group (for age, BMI) of 40 patients on Atkin's diet.

**Results:**

Two comparable groups of obese patients were studied, each group consists of 40 female patients, mean age in group A was 27 years (20–39 years) with mean body weight 90 kg (80–100) and mean body mass index 36 (31–39.9) who were treated with insertion of BIB. While mean age in group B was 29 years (20–39 years) with mean body weight 91 kg (80–102) and mean body mass index 36.5 (31–39.9). Statistically important changes occurred in the quality of life of the patients after either method of treatments to different degrees (p-value 0.005917).

**Conclusion:**

The patients lost more weight after insertion of intra-gastric balloon up to 35 kg of body weight, while the patients on Atkins diet lost up to 20 kg body weight. This results in statistically significant improvement of most aspects of QOL. Especially in Feeling happier, more satisfaction with the new body image, Improvement of self-esteem, encouraged for more regular exercises, less nervous, embarrassed less by unimportant matters, have less negative thoughts, and the craving of foods decreased remarkably.

## Highlights

• Low-carbohydrate, high-protein, high-fat diets have become increasingly popular.
• Low-carbohydrate diet produced a greater weight loss than did the conventional diet.
• The goal is the possibility of weight loss with the improvement in the quality of life.
• Bioenterics intragastric balloon reduces more weight and BMI versus modified Atkins diet.

## 1 Introduction

Obesity is a chronic disease and among the most severe health problems worldwide, its prevalence is dramatically increasing, and this increase represents a leading public health problem [1].

According to the World Health Organization (WHO), one billion adults throughout the world are overweight, with a body mass index (BMI in kg/m2) above 25. Of these, at least 300 million are considered obese (BMI 30 and more) [2,3].

Obesity affects every aspect of the life of the patients, health-related aspects, albeit affects psychological, spiritual, and physical aspects of the life [4]. Weight loss is a major concern, surveys consistently show that most adults are trying to lose or maintain weight [5].

Low-carbohydrate, high-protein, high-fat diets have become increasingly popular, availability of diet books may promote this approach. The book titled Atkins diet, originally published in 1973 and again in 1992 and 2002, may make the Atkins diet the most popular of these diets [6,7].

In the literature, one may find several articles stating that“ the low-carbohydrate diet produced a greater weight loss than did the conventional diet for the first six months” [8] after dieting.

Another option is the implantation of BioEnterics^®^ Intragastric Balloon (BIB^®^) System (BIB) which is a relatively new method for treating obesity, becoming quite popular worldwide Since 1998 [9], it seems to have a possibility of achieving the weight loss with the consequences of improvement in obesity-related comorbid conditions and quality of life. While it is a simple and easy option [1], some authors claim that it has the characteristics of an “ideal gastric balloon” [10], especially in patients whose obesity is not severe enough to warrant surgery [9].

“Health-related quality of life (HRQOL) of obese patients” [11], before and after weight loss by insertion of BioEnterics Intra-gastric Balloon is studied widely. But the quality of life like mood, satisfaction with relationships, achieved goals, self-concepts, and self-perceived ability to cope with one's daily life is “not studied on a wide scale” [[12], [13], [14], [15], [16], [17], [18]].

The QOL Scoring is a valid instrument for measuring the quality of life across patient groups and cultures and is conceptually distinct from health status or other causal indicators of quality of life [1,19,20].

The current work is an evaluation of the effect of obesity on different aspects of life, and to evaluate the influence of weight loss after BIB insertion or Atkins diet on the quality of life regarding mood, satisfaction with relationships, achieved goals, self-concepts, and self-perceived ability to cope with one's daily life.

## 2 Patients, materials, and methods

A prospective randomized study, from a total of 180 patients, 80 patients were selected to enroll in the work, over a period of 4 years from 2008 to 2012 in Hatwan private hospital and the private clinic, the closing date was 1st January 2013.

Approval for the current work was obtained from the Ethics Committee of University, College of Medicine (No.18 on 23 August 2013). The work has been reported in line with the STROCSS criteria (22). Registered in research registry under number (Research Registry UIN: 4431). The study was designed as a descriptive longitudinal study conducted on 40 patients who underwent intra-gastric balloon insertion compared to a matched group (for age, BMI) of 40 patients on Atkin's diet.

For the collection of the required data, every patient was interviewed face-to-face, by a junior doctor who was working in the Hatwan Private Hospital, to sign informed consent, and to fill an originally-designed questionnaire in English translated to the Kurdish language. It is composed of demographic, medical, and biological data, besides the important aspects of quality of life, before the management and 6 months after completing their management. The statistical evaluations were all done with SPSS version 21. Chi-square test adjusted for clinical characteristics: age groups, BMI, waist circumference, changes in QOL, and all statistical tests were assessed at the conventional 0.05 level of significance.

Included patients were divided into two comparable groups.

1. Group A; 40 single obese female patients with mean body weight 90 kg (80–100) and mean Body mass index (BMI) 36 (31–39.9) treated with insertion of Bioenterics intra-gastric balloon (BIB).
2. Group B; 40 single obese female patients with mean body weight 91 kg (80–102) and mean BMI 36.5 (31–39.9), subjected to modified Atkins diet for 6 months, each patient in either group was followed up monthly for 18 months.

All single females from 20 to 40 years of age, have BMI 30 to 39.9, patients, who were otherwise healthy, those neither have psychological problems nor taking psychotropic drugs, and patients with no history of insertion of Bio-enteric intra-gastric balloon (BIB) or any form of bariatric surgery were included, as well as those with no peptic ulcer diseases, patients were no binge eaters.

All patients who were on steroid, and those not committed to the diet, those with peptic ulcer diseases, and binge eating patients were excluded.

The baseline assessment included medical history, physical examination, anthropometric status (body weight/height, BMI), blood pressure, electrocardiogram, laboratory diagnostics (complete blood count, coagulation, hepatic profile, renal profile, lipid profile, fasting blood glucose levels, glycosylated hemoglobin—HbA1c, and CRP) and transabdominal ultrasound, upper gastrointestinal endoscopy. While patients in group A were also subjected to spirometry, chest radiography.

Body Weight and height were measured with a calibrated scale and a wall-mounted stadiometer while the subjects were wearing light clothing and no shoes at the start and during each visit.

At first upper gastrointestinal tract checked by esophagogastroduodenoscopy (OGD), then The Bio-Enteric Intra-gastric Balloon (BIB) (Allergan Incorporation, Irvine, California, CA, USA) was inserted under sedation (1 mg intravenous Midazolam injection) by an anesthesiology team. The BIB was filled with a volume of 600-ml sterile saline containing 10 ml methylene blue (10%). The position and size of the inserted intra-gastric balloon were verified by abdominal radiography and ultrasound.

All patients were put on (intravenous ondansetron 8 mg 8 hourly) for the first three days and a proton pump inhibitor (40 mg Lansoprazole, single oral daily dose) for the first four weeks.

Each patient was asked to visit as an outpatient; after 7 days and 14 days, followed by monthly controls. The balloon was removed after 6 months as recommended by the manufacturer.

Any patient who refused balloon or was unable to pay for the balloon insertion and removal was told the details about the Atkin's Diet. There is a shortage of most of the Atkins foods and formulas here; this is why Atkins meals were modified to suit the patients and the area in the following manner; (not to take the following items; Sugar, sweet foods and fruits, rice, potato, and white bread, allowing just 100 calories in the form of one green apple weighing 190 gm).

## 3 Results

Two comparable groups of obese patients were studied, each group consists of 40 female patients, mean age in group A was 27 years (20–39 years) with mean body weight 90 kg (80–100) and mean BMI 36 (31–39.9) who were treated with insertion of BIB. While mean age in group B was 29 years (20–39 years) with mean body weight 91 kg (80–102) and mean BMI 36.5 (31–39.9), Table 1.

**Table 1:** Number of the patients in each extent of excess body weight loss in both groups A and B.

Groups	Excess body Weight loss (10–15 kg)	Excess body Weight loss (16–20 kg)	Excess body Weight loss (21–25 kg)	Excess body Weight loss (26–30 kg)	Excess body Weight loss (31–35 kg)
A	4	6	7	4	**19**
B	30	10	0	0	0
P value	0.00010 significant		0.00001 significant		

They lost weight in the first 6 month of treatment as shown in Table 1, with the highest loss of body weight in group A, as 19 (47.5%) of them lost 33 kg (P-value 0.00001), while in the group B, only 10 patients (25%) lost 17 kg of their body weight as highest loss of weight (P-value 0.00010) as shown in Table 1.

During the first interview with the patients before starting the treatment to fill the questionnaire, most of the patients admitted having at least one meal outdoor weekly and fewer subjects two outdoor meals weekly look to Table 2, all the patients were literates, with a diploma ranging from high school up to Ph.D. grades.

**Table 2:** Aspects of QOL of the obese patients in both groups A and B before starting of the treatments.

Aspects of QOL	Scoring							
	Not at all		Just a little		Not so much		Much	
Groups	**A**	**B**	**A**	**B**	**A**	**B**	**A**	**B**
Feeling happy	39	37	0	1	1	1	0	1
Satisfaction with the body image	40	40	0	0	0	0	0	0
Feel high self-esteem	25	27	10	9	4	4	1	0
The feeling of Sexually attractive	2	2	18	10	3	12	17	16
Wish to have better or more regular exercise	8	9	15	13	1	3	26	25
Spending more time with friends	33	33	7	6	7	0	0	1
Nervousness, embracing by unimportant matters	0	0	0	0	0	0	**40**	**40**
Hearing sarcasm or teasing by others	1	1	0	1	0	0	**39**	**38**
Negative thoughts	1	2	2	2	2	1	**35**	**35**
Satiety decreased	40	25	0	13	0	2	**0**	**0**

All the patients admitted to the importance to losing weight and have better body shape, while one third claimed that it is important to be sexually attractive.

A large number of the patients were embarrassed by prompt discussions in the family because of being obese and jokes and sarcasm and teasing from others they face daily. They became more apprehensive and embraced by unimportant matters. Three quarters were afraid from what they hear and see from mass media about risks of obesity and how their weight and sight became an obstacle for obtaining the job they like an obstacle for success in their jobs as shown in Table 2.

All the patients admitted that their obesity narrowed their social circle. Most felt that they have less opportunity to spend time with friends, felt uneasy to show their body in swimming, sports.

About three quarter were withdrawn, feeling lonely, and have negative self-thoughts. One patient in (group A) had the history of attempted suicide with three patients in group B having suicidal thoughts.

After 6 months from treatment with BIB or treatment with modified Atkins diet, Table 3, every patient was interviewed to fill the 2nd questionnaire, regarding changes in her quality of life after the treatment and losing weight.

**Table 3:** Aspects of QOL of the obese patients in both groups A and B after 6 months of the treatments.

Aspects of QOL	Scoring							
	Not at all		Just a little		Not so much		Much	
Groups	A	B	A	B	A	B	**A**	**B**
Feeling happy	0	0	0	0	0	7	**40**	**33**
Satisfaction with the new body image	0	3	0	6	1	1	**39**	**30**
Improved self-esteem	0	1	0	0	0	2	**40**	**37**
The feeling of Sexually attractive	0	0	0	0	0	0	**40**	**40**
Have better or more regular exercise	0	0	0	2	0	4	**40**	**34**
Spending more time with friends	0	0	0	2	2	3	**38**	**35**
Nervousness, embracing by unimportant matters	38	33	1	2	1	2	**0**	**3**
Hearing sarcasm or teasing by others	40	36	0	1	0	3	**0**	**0**
Negative thoughts	39	34	1	1	0	1	**0**	**4**
Satiety decreased	0	0	0	13	0	2	**40**	**25**

All the patients in group A, who were treated by BIB, lost greater body weight versus patients in group B, all admitted that they feel happy, sexually attractive, have more regular exercise, and their self-esteem was noticeably improved and their craving foods were significantly decreased.

All the patients in group A and B stated that they heard no more jokes, sarcasm about them and were not teased, Table 3.

The most changed aspects of QOL were compared in the patients treated with BIB, (group A) with those patients treated with modified Atkins diet (group B), Table 4.

**Table 4:** Comparison the highest respond reflecting changes QOL of the obese patients in both groups A and B after completion of the treatments.

Aspects of QOL	Comparison by highest respond		P value
	Group A Who answered by much	Group B Who answered by much	
Feeling happy	**40**	**33**	**0.005917**
Satisfaction with the new body image	**39**	**30**	**0.0036889**
Improved self-esteem	**40**	**37**	**0.079362**
The feeling of Sexually attractive	**40**	**40**	**0.4936709**
Have better or more regular exercise	**40**	**34**	**0.0113772**
Spending more time with friends	**38**	**35**	**0.0217377**
Nervousness, embracing by unimportant matters	**0**	3	**0.079362**
Hearing sarcasm or teasing by others	**0**	0	**0.4936709**
Negative thoughts	**0**	4	**0.0414411**
Increased satiety	**40**	25	**0.0143772**

## 4 Discussion

The diet used was modifying the Atkins diet, to suit our custom of food and available foods in our locality. We abandoned other items besides sugar, because of the fact that they contain high calorie and carbohydrate content; rice, white bread, and potato, which contains (365, 327 and 77-kilocalories for each 100 gm) respectively [11].

Sweet foods were abandoned because of their high contents of sucrose, glucose or fructose [21,22,23,24] which all increases weight energy intake [24,25]. Fructose as it is not, stimulates insulin secretion from beta cells; leads to increased caloric intake and obesity [22,24,26,27], and reduction in added sugar is one means to achieve a reduction in energy density [26], and significantly alter hepatic insulin sensitivity and lipid metabolism compared with similar amounts of glucose [23].

The allowance for energy from carbohydrate was 100 kcal equal to the content of the 190 gm apple [23]. WHO has suggested that added sugars should provide no more than 10% of dietary energy [28], and a prudent upper limit of intake is half of the discretionary calorie allowance, which is not more than 100 calories per day and for most American men is no more than 150 kilocalories per day from added sugars [27].

Mechanism of action of BIB is similar to that of gastric restrictive operations, i.e. a reduction in gastric volume [26,29,30,31], which remains for food intake, also has a satiating effect [32]. Even if BIB was filled with a volume of (400 mL) it will reduce food intake, but to obtain maximal weight loss without complications we filled the balloon with 600 cc (9, 30), saline containing 10 cc of 10% methylene blue.

This volume of the balloon will cause more stomach distension, which will increase the release of Leptin [32,33] which are associated with subjective hunger ratings and with lower food intake, and reduces the secretion of Ghrelin [33,34,35].

Although QOL improved in both groups A & B, the results reinforce the benefit of BIB over Atkins in improving the different aspects of QOL as shown in Table 3, findings showed that in all patients (100%) of group A, they admitted happiness and satisfaction with the results, their self-esteem improved noticeably, found themselves to be sexually attractive, (P-value 0.0113772) and have regular exercises (P-value 0.0113772), and their satiety meaningfully diminished (P-value 0.0143772), while only (62.5–92.5%) from group B felt this way see Table 3 and Table 4.

Merely in two aspects all the patients from (group B) like (group A), improved significantly, these were feeling sexually attractive and no more sarcasm and teasing from others. We could explain these differences, it is due to that the patients in group A lost substantial weight (P-value 0.00001), while the patients in group B, were “with minor weight loss” [32] were less positive for improvement in QOL, look to Table 4.

In the results, the primary outcome as changes in overall QOL, the peak improvement in weight and QOL were noted after 6 months and one year from the starting of the treatments [36,37], this result is going with the findings in other studies [38].

This may means that the 6–12 months is enough time in evaluating the extent of weight loss and its effect on QOL of any sliming treatments.

The results correlated lowering of BMI after the treatment, with improvements in QOL, is in line with other studies [[38], [39], [40], [41], [42], [43]].

One of these changes in both groups but for different levels is satiety, which was measured by “two ways, asking the patient to answer subjectively and amount of food taken in each meal”, the different level of satiety was noticed between patients of (group A) 100% versus patients of (group B) 62.5%, (p-value 0.0000). This may be explained, by the presence of BIB in the stomach, causing stomach distension which may result in satiety, this seen in all the patients in (group A). There is a study in the literature comparing (BIB plus diet) to (diet) alone regarding amount of weight loss, but not QOL [9,33], while the changes in QOL in a psychological and self-esteem, in group A, is much higher than group B and higher than the results of a study by Y. Mart [40]. BIB reduces more weight and BMI in 6 months in comparison to modified Atkins diet and results in statistically significant improvement in most aspects of QOL.

## 5 Limitations

Shortage of the Atkins formula and foods in the area, if original Atkins was followed, with special foods and formula, the results might be different, both in the amount of excess body weight and improvement of aspects of QOL. The small number of patients, is also considered as a limitation.

## 6 Conclusion

The patients lost more weight after insertion of intra-gastric balloon up to 35 kg of body weight, while the patients on Atkins diet lost up to 20 kg body weight. This results in statistically significant improvement of most aspects of QOL. Especially in Feeling happier, more satisfaction with the new body image, Improvement of self-esteem, encouraged for more regular exercises, less nervous, embarrassed less by unimportant matters, have less negative thoughts, and the craving of foods decreased remarkably.

## Ethical approval

Approved by Ethics committee of the College of Medicine-University of Sulaimani No. No.18 on 23 August 2013.

## Sources of funding

No any sources of funding for the research.

## Author contribution

Study design: Hiwa Ahmed.

Data collection: Rajan F. Ezzat.

Statistical analysis: Dr. Abdulfatah Muhamad.

Writing the first draft of the manuscript: Hiwa Ahmed.

Critical review and approval of manuscript: Hiwa Ahmed, Rajan F. Ezzat.

## Conflicts of interest

No conflicts of interest to declare.

## Research registration number

research registry UIN 4431.

## Guarantor

Hiwa Ahmed.

## Provenance and peer review

Not commissioned, externally peer reviewed.
